# Thy-1 as an Integrator of Diverse Extracellular Signals

**DOI:** 10.3389/fcell.2019.00026

**Published:** 2019-02-25

**Authors:** James S. Hagood

**Affiliations:** Division of Pulmonology, Department of Pediatrics, University of North Carolina at Chapel Hill, Chapel Hill, NC, United States

**Keywords:** Thy-1, signaling, mechanotransduction, stem cells, viral entry, extracellular vesicles

## Abstract

Thy-1 was discovered over 50 years ago, and in that time investigators from a broad variety of fields have described numerous and heterogeneous biological functions of Thy-1 in multiple contexts. As an outwardly facing cell surface molecule, it is well positioned to receive extracellular signals; previously reviewed studies have confirmed an important role in cell-cell and cell-matrix adhesion, cell migration, and regulation of outside-in signaling. More recent studies reviewed here expand the repertoire of Thy-1 effects on signaling pathways, and reveal novel roles in mechanotransduction, cellular differentiation, viral entry, and extracellular vesicle binding and internalization. All of these studies contribute to understanding Thy-1 as a context-dependent integrator of a diverse range of extracellular information, and provide impetus for further studies, some of which are suggested here.

## Introduction

More than 50 years after its original description as a lymphocyte marker (Reif and Allen, 1964), Thy-1 (CD90) remains an enigmatic molecule. It is expressed on the surface of numerous and diverse cell types, and confers varied effects on cell phenotype, depending on context (reviewed in Bradley et al., 2009). Accordingly, the functions of Thy-1 have been studied in a broad range of fields, including immunology, neurobiology, cancer, stem cell biology, tissue remodeling and aging, and have been the focus of multiple excellent reviews (Haeryfar and Hoskin, 2004; Rege and Hagood, 2006; Herrera-Molina et al., 2013; Leyton and Hagood, 2014; Kumar et al., 2016). In this mini-review, we consider some recent studies that reveal novel aspects of Thy-1 biology that have not been extensively studied, attempt to synthesize these within an emerging view of this molecule, and suggest future areas of exploration.

## Thy-1 and Intracellular Signaling

The effects of Thy-1 on cell signaling have been intriguing. An excellent recent review by a pioneer in the field details the signaling effects of Thy-1 relative to its abundance and location on cell surfaces (Morris, 2018). Thy-1 does not function exclusively in a single classic receptor/ligand-type interaction, but additionally can interact with a number of molecules either within the membrane of the same cell (cis) or heterotypically with molecules on the surface of another cell (trans), and in the latter case can affect signaling within the Thy-1-bearing cell or the interacting cell (Herrera-Molina et al., 2013). Known interacting partners include a number of integrin heterodimers, heparin sulfate proteoglycans (e.g., syndecan 4), and some G-protein coupled receptors (Leyton and Hagood, 2014). Interactions of Thy-1 with integrin signaling, particularly though αv heterodimers and Src family kinase (SFK) activation are perhaps the best characterized, however much remains to be learned. An important challenge has been understanding how Thy-1, as a gycosylphosphatidyl inositol (GPI)-anchored molecule which lacks a transmembrane domain, affects the activation of intracellular signaling molecules such as Src family kinases (SFKs). A recent study carefully dissected the molecular interactions by which Thy-1/αvβ3 interaction regulated SFK activation and cytoskeletal rearrangement in the context of neuron-astrocyte communication (Maldonado et al., 2017). Astrocyte αvβ3 interacts with neuronal Thy-1 in trans to induce neurite retraction. The authors applied two-channel, super-resolution stimulated emission depletion (STED) microscopy combined with single-molecule tracking to show that This interactions slows movement of Thy-1 in the neuronal membrane, promoting formation of aggregates of Thy-1 composed of smaller nanoclusters. These clusters include C-terminal Src kinase (Csk)-binding protein (CBP), a transmembrane scaffolding protein that had previously been shown to confine Thy-1 within lipid raft microdomains (Chen et al., 2009). Cytoplasmic Csk associated with CBP-Thy-1 clusters phosphorylates and inactivates Src, displacing it from these clusters. Interestingly, inactive Src associates with Thy-1 in separate clusters distinct from the Thy-1/CBP/Csk complex. Downstream, inactive Src results in activation of p190RhoGAP, which in turn results in activation of RhoA, resulting in cytoskeletal alterations leading to neurite retraction. A similar pathway downstream of Thy-1/integrin interaction in cis had been previously demonstrated in fibroblasts (Fiore et al., 2015), in which it regulates cell adhesion, cytoskeletal organization, and myofibroblastic differentiation.

Thy-1 interacts with other, non-integrin signaling pathways to modulate cellular phenotype. In fibroblasts, Fas ligand promotes Thy-1/Fas interactions in lipid rafts to promote apoptosis (Liu et al., 2017). This pathway appears important to the resolution of fibrosis, as Thy-1^-/-^ mice fail to resolve fibrosis following bleomycin-induced lung injury, associated with the persistence of apoptosis-resistant myofibroblasts. A study in hepatocellular carcinoma cells showed that the presence of Thy-1, which is a cancer stem cell marker in these tumors, is correlated with enhanced Notch signaling (Luo et al., 2016). This study did not manipulate Thy-1 expression to demonstrate whether this effect is direct or indirect, but this is the first demonstration of an association of Thy-1 with Notch signaling. The broader role of Thy-1 in cancer is complex, as it has been shown to both promote tumorigenesis and to function as a tumor suppressor; this conundrum has been recently reviewed (Kumar et al., 2016). In the context of liver fibrosis Thy-1 was recently found to interact with TGFβRI, indicating a novel mechanism whereby Thy-1 affects TGF-β1 signaling and myofibroblast differentiation (Koyama et al., 2017). Previously, Thy-1 had been shown to inhibit latent TGF-β1 activation in an αv integrin-dependent manner (Zhou et al., 2004, 2010). In liver portal fibroblasts, Thy-1 binding to TGFβRI was shown to be disrupted by mesothelin, indicating two additional novel molecular interactions not previously described for Thy-1 (Koyama et al., 2017). Besides the known integrin- and heparin-binding motifs in the Thy-1 sequence, the mechanisms for many of Thy-1’s molecular interactions are not known.

## Thy-1 and Mechanotransduction

In addition to responding to chemical signals, most living cells alter their phenotype in response to mechanical stimuli from the external environment. This involves a complex series of molecular interactions from the cell surface to the nucleus (Wang, 2017). Because Thy-1 interacts with numerous integrins and modulates signaling pathways (SFKs, Rho kinases) involved in mechanotransduction, it is well positioned to participate in cellular responses to mechanical cues. Indeed, Thy-1 was found to participate in mechanical signaling in melanoma cells, where it forms a trimolecular complex with α5β1 integrin and syndecan 4 (Fiore et al., 2014). Interestingly, this molecular complex displays binding characteristics of what has been termed “dynamic catch bonds,” characterized by rapid bond stiffening and increased binding affinity when force is applied to the bond. More recently, in lung fibroblasts, Thy-1 has been shown to function as a mechanosensor, in that knockdown of Thy-1 results in an inability of fibroblasts to modulate signaling and cell phenotype in response to either increased or decreased matrix stiffness (Fiore et al., 2018). This mechanosensing function is accomplished through Thy-1 interacting with αvβ3 integrin, altering its avidity for ECM binding, while localizing SFKs necessary for downstream mechanosignaling. Loss of Thy-1 in the context of lung injury thus leads to increased integrin activation even in soft provisional ECM, which promotes strain stiffening of matrix, further integrin activation, and progressive fibrosis. *In vivo*, absence of Thy-1 leads to non-resolving fibrosis in a mouse model of lung injury (Fiore et al., 2018). A recent study used molecular force spectroscopy to characterize the mechanical properties of the Thy-1/αvβ3 integrin interaction at the single-molecule level using purified proteins (Burgos-Bravo et al., 2018), and applying mathematical modeling to characterize the slip-bond interaction between these two molecules.

## Thy-1 and “Stemness”

Thy-1, also known as cluster of differentiation (CD)90, has long been recognized and broadly utilized as a marker of hematopoietic and mesenchymal stem cells. Until recently, however, any mechanistic role in the stem cell phenotype has not been appreciated. Reprogramming of differentiated fibroblasts into induced pluripotent stem cells (iPSCs) using a limited number of transcription factors is one of the major milestones in 21st century biology (Takahashi and Yamanaka, 2006). A study exploring the role of microRNAs (miRs) in fibroblast reprogramming found that after exposure of murine embryonic fibroblasts (MEFs) to the pluripotency-inducing “four factors” (4F), loss of Thy-1 (CD90) was a strong marker of early reprogramming (Li et al., 2014). However, when 4F-infected MEF were sorted into Thy-1^+^ and Thy-1^-^ subpopulations, fully pluripotent iPSCs were highly enriched in the Thy-1^-^ subpopulation. Loss of Thy-1 was associated with increased expression of miR-135b, which targets a number of ECM-associated genes, including genes involved in TGFβ1 signaling. MEFs which retained Thy-1 expression after 4F infection did not induce miR-135b and retained expression of ECM-associated genes, and had a more limited, multipotent MSC-like phenotype compared to the truly pluripotent Thy-1^-^ iPSCs. This very interesting set of findings suggests that Thy-1 may function as a barrier to pluripotency, and that a mesenchymal Thy-1^+^ phenotype is associated with a microRNA and gene expression profile associated with regulation of ECM-related genes.

Even in MSCs, for which Thy-1 is often used as a marker, its role with regard to differentiation potential is complex. An interesting recent study showed that in human MSCs cultured from numerous sources, lentiviral suppression of Thy-1 increases the capacity for osteoblastic and adipogenic differentiation (Moraes et al., 2016). Complicating matters, MSCs isolated from Thy-1^-/-^ mice were found to have decreased osteoblastic differentiation and increased adipocyte differentiation compared to those from WT mice, consistent with findings of increased adiposity and decreased bone density in Thy-1^-/-^ mice (Picke et al., 2018). These findings suggest that the relationship of Thy-1 expression and mesenchymal differentiation is complex and context-dependent, and that Thy-1 may have many different roles in the pathway from pluripotent stem cells to fully differentiated cells. ECM composition and stiffness are known to affect “stemness,” and the roles of Thy-1 as a mechanotransducer and sensor of ECM, as well as a regulator of TGF-β signaling within this context, has yet to be fully elucidated.

## Thy-1 and Viral Entry

The co-evolution of viruses and animal cells has led to a complex array of receptors and mechanisms for viral entry, with strong significance for viral pathogenesis, transplantation, and virus-mediated gene targeting (Baranowski et al., 2003). Many of the viral entry pathways include molecules which regulate cell-cell and cell-matrix interactions, such as integrins. Thus it is not surprising that Thy-1 is involved in this area of biology as well. human cytomegalovirus (HCMV) is a highly prevalent virus that is responsible for human birth defects and organ transplant complications. Thy-1 has recently been shown to play a critical role in entry of HCMV into certain cell types, and more recently this has been demonstrated to involve regulation of clathrin-independent micropinocytosis (Li et al., 2016). Further understanding of this likely primitive mechanism may lead to development of novel inhibitors of viral infection.

## Thy-1 and Extracellular Vesicles

Membrane-bound vesicles are critical components of intracellular trafficking and signaling. In the past decade there has been tremendous growth in understanding of the roles of secreted extracellular vesicles (EVs), such as exosomes, larger microvesicles, and apoptotic bodies. We had previously demonstrated release of Thy-1 from fibroblasts in response to inflammatory stimuli, and antibody studies indicated that Thy-1 was likely released in a membrane-bound fraction (Hagood et al., 2005; Bradley et al., 2013). Subsequently we demonstrated that MSCs secrete large numbers of EVs, most of which have Thy-1 on the EV surface. These MSC-derived EVs bind to and are internalized by lung fibroblasts, and modulate myofibroblastic differentiation and response to TGFβ1 (Shentu et al., 2017). Antibody-mediated blocking of Thy-1 or β3/β5 integrins prevents EV binding and uptake, as does shRNA-mediated downregulation of Thy-1 and integrins on recipient cells. These findings indicate that Thy-1, either on the EV surface or the recipient cell surface, interacts with integrin on the opposite (cell surface or EV) to facilitate binding, uptake, and delivery of EV content, predominantly miRNA. This is likely similar to the mechanisms for Thy-1-mediated viral entry discussed above. The role of Thy-1 on intracellular vesicles is unknown, but it has been found in non-caveolar vesicles and neuronal synaptic vesicles (Jeng et al., 1998; Yao et al., 2009).

## Summary and Insights

**FIGURE 1:**
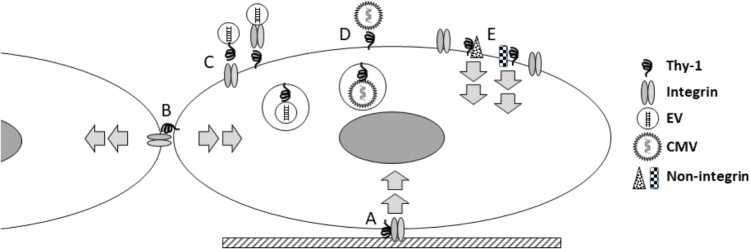
Thy-1 integrates diverse extracellular signals. Thy-1 has been known to participate, via interaction with integrins, in transducing signals from the extracellular matrix **(A)**; more recently this understanding has expanded to include mechanical signals. This type of signaling involves Thy-1 interacting in cis with other molecules within the same cell membrane. Thy-1 is also known to interact in trans with molecules in other cells, in mediating cell-cell interactions **(B)**, with signaling effects in both cells. More recently, Thy-1 has been shown to facilitate binding and internalization of extracellular vesicles (EV; **C**) and cytomegalovirus (CMV; **D**). Increasingly, Thy-1 has been shown to interact with a growing number of non-integrin signaling partners **(E)**. Because the signaling associated with Thy-1 regulates many fundamental cellular processes (stemness, differentiation, migration, and survival), ongoing studies to better understand the molecular mechanisms involved will continue to yield important biological insights about how cells integrate extracellular information.

How do these seemingly disparate new findings inform our current view of Thy-1? The last half century has produced a substantial body of work on this molecule, during which its roles in a large number of biological fields have become apparent. What insights can be gained from what is known about Thy-1, and what are important questions for future studies? First, Thy-1 is a primitive molecule, conserved to the level of chordates (Mansour et al., 1987), and it is involved in very basic functions (cell-ECM and cell-cell adhesion) important in multicellular biology. The role of Thy-1 in viral entry, EV binding, and signaling all support a broader role for Thy-1 in receiving and processing information from the environment (Figure 1). It may be enlightening to consider how Thy-1 might regulate trafficking and processing such information intracellularly. For example, does Thy-1 affect intracellular vesicular functions? Secondly, Thy-1 regulates cell phenotype changes in response to external stimuli, even fundamental characteristics such as pluripotency, differentiation and survival. Understanding the mechanisms by which it does so may yield novel insights into these fundamental processes. Third, the nature of Thy-1’s effects seem critically dependent on context, so that what Thy-1 does in one tissue or cell type cannot be simply extrapolated to others. Finally, many but not all of Thy-1’s effects involve interaction with integrins, but it is important to remember that Thy-1 also interacts with a growing number of other molecules, and that it may function as part of multi-molecular complexes which affect several important intracellular signaling cascades.

Many unanswered questions remain. Little is known, for example, about how the mode of anchorage of Thy-1 (i.e., its particular GPI anchor) affects its function. Is it merely by regulating its proximity to integrins and other surface molecules, or is the lipid moiety of Thy-1 itself able to interact with other molecules to affect signaling? How important are other post-translational modifications, most notably glycosylation, in some of the seemingly discordant effects of Thy-1 in different contexts? Many of the downstream effects of Thy-1 involve cell phenotype and “identity.” These are likely regulated at the level of transcriptional control and epigenetic modifications. Does Thy-1 directly affect nuclear machinery, or are all its effects secondary to its effects on cytoskeletal rearrangements and signaling cascades? With improved understanding of mechanisms comes the ability to target specific aspects of Thy-1 function. However, the translational potential of manipulating Thy-1 remain unexplored. Perhaps the next half century will see important breakthroughs as a result of additional careful study of this enigmatic integrator of extracellular information.

## Author Contributions

JSH reviewed the relevant literature and wrote the manuscript.

## Conflict of Interest Statement

The author declares that the research was conducted in the absence of any commercial or financial relationships that could be construed as a potential conflict of interest.
